# Mapping links between childhood maltreatment and biopsychosocial health in pregnancy: a network analysis

**DOI:** 10.1007/s00737-026-01749-4

**Published:** 2026-08-19

**Authors:** Jana Katharina Ray, Anna-Lena Zietlow, Julia Ditzer, David Kolar, Monique Stenzel, Julia Téoule, Marc Sütterlin, Martin Lablans, Barbara Filsinger

**Affiliations:** 1 Department of Gynecology and Obstetrics, Medical Faculty Mannheim of University of Heidelberg, Mannheim, Germany; 2 Chair of Clinical Child and Adolescent Psychology, TU Dresden, Dresden, Germany; 3 Department of Psychology, University of Regensburg, Regensburg, Germany; 4 Complex Medical Informatics, Medical Faculty Mannheim of University of Heidelberg, Mannheim, Germany; 5 Federated Information Systems, German Cancer Research Center, Heidelberg, Germany; 6 Professorship for Obstetrics and Mother-Child Health, Ludwigshafen University of Business and Society, Ludwigshafen am Rhein, Germany

**Keywords:** Abuse and neglect, Adverse childhood experiences, Prenatal risk, Transition to parenthood, Perinatal depression, Social support

## Abstract

**Purpose:**

Child maltreatment experiences may impact both mental and physical health in pregnancy and are associated with increased interpersonal difficulties. However, facets of psychosocial and physical health are interrelated, making it difficult to identify effects of maltreatment experiences. Therefore, this study aims to improve our understanding of how experiences of childhood maltreatment are associated with perinatal bio-psycho-social health employing an exploratory network approach. The analysis focused on whether there are differential effects of maltreatment subtypes, namely physical, sexual and emotional abuse as well as physical and emotional neglect.

**Method:**

*N* = 131 pregnant women (gestational age of 10–20 weeks) answered questionnaires on their own child maltreatment experiences, depressive and anxiety symptoms, maternal-foetal attachment, social support and couple relationship quality. Additionally, medical information was collected from their maternity passports. We estimated a regularized partial correlation network to model the associations between these variables.

**Results:**

Maltreatment experiences were associated with lower prenatal social support and higher prenatal depression scores, but not with pregnancy-related anxiety, maternal-foetal attachment and prenatal relationship quality. A link between maltreatment and medical risk emerged only in a reduced network model including only emotional child maltreatment subtypes, but not physical or sexual maltreatment. Among subtypes, emotional abuse and emotional neglect emerged as most central in the network.

**Conclusion:**

Emotional abuse and neglect experiences may function as risk factors for biopsychosocial health in early pregnancy. Thus, maltreatment experiences should be examined carefully in medical history. Their effects, especially regarding perceived social support, need to be addressed in prenatal care by gynaecologists and midwives.

**Supplementary Information:**

The online version contains supplementary material available at https://doi.org/10.1007/s00737-026-01749-4.

Pregnancy and the transition to motherhood are pivotal periods for women’s health. Life transition phases are typically accompanied by heightened vulnerability (Daskalakis et al. 2013), as evident in the substantial prevalence of mental health problems among pregnant women worldwide (Nielsen-Scott et al. 2022; Woody et al. 2017). An important transdiagnostic risk factor complicating transitions is a history of childhood maltreatment (CM). A widely accepted framework distinguishes between five maltreatment subtypes: emotional, physical, and sexual abuse, as well as emotional and physical neglect (Herrenkohl 2005; Manly 2005). Across these subtypes, childhood maltreatment has profound effects on individuals’ physical (Chen et al. 2023; Danese and Tan 2014; Kerr et al. 2021) and mental health (Green et al. 2010; Li et al. 2016; Zeanah and Humphreys 2018) around the globe. These effects unfold through several mechanisms, such as impacted stress management and emotion regulation (Gruhn and Compas 2020; McLaughlin et al. 2015; Miu et al. 2022) – coping mechanisms highly required in critical life periods like pregnancy. Consequently, pregnancy may pose mental and physical health challenges even for those who have so far successfully coped after CM.

Recent studies investigating effects of CM on pregnancy and transition to motherhood mainly examined its effects on perinatal mental health, with CM being one of the most important predictors of perinatal depression and anxiety (Christie et al. 2018; Racine et al. 2021). However, the consequences of CM extend far beyond mental health. CM is associated with both behavioural (Halpern et al., 2018) and epigenetic alterations (Parade et al., 2021), which may adversely affect physical health. During pregnancy, this may manifest as increased medical risks, as particularly preterm birth, low birth weight, and gestational diabetes have been found in those affected by CM (Frederickson et al. 2024; Kern et al. 2022; Mamun et al. 2023). Additionally, CM can substantially impair emotion processing (Ditzer et al. 2023), potentially increasing vulnerability to mental health problems and difficulties in interpersonal relationships later in life. Consistent with this, adults with a history of CM report lower relationship quality and higher levels of conflict in romantic partnerships (Schütze et al. 2020; Zamir 2022). Finally, CM seems to affect the developing interpersonal relationship between mother and infant by increasing depressive symptoms and thereby disrupting attachment processes (Souch et al. 2022) which begin during pregnancy (Cuijlits et al. 2019).

Despite this clear evidence for the impact of CM on women’s perinatal mental, physical and social health, understanding the complex interplay between perinatal factors and CM remains a key challenge. Risk factors such as depression, anxiety, medical complications, and relationship difficulties do not occur in isolation but rather influence each other. For instance, lower relationship quality is associated with maternal perinatal depression (Pilkington et al. 2015; Ray et al. 2023). Similarly, anxiety is higher in pregnant individuals who experience medical pregnancy complications (Abrar et al. 2020) and has consequences for the mother-child relationship (Zietlow et al. 2019). Maternal-foetal attachment is crucial for postnatal outcomes, and is influenced by partner support (Cuijlits et al. 2019). Another research gap is understanding the different effects of the subtypes of CM on prenatal health. While survivors of CM often experienced multiple forms of abuse or neglect simultaneously (Warmingham et al. 2019), each subtype represents a unique set of experiences that may be linked to specific outcomes. While it has been demonstrated that emotional abuse and emotional neglect are particularly associated with depression in later life (Humphreys et al. 2020), other perinatal factors may be more closely associated with other CM subtypes.

In conclusion, a comprehensive understanding of how experiences of CM affect pregnant individuals requires an integrated biopsychosocial framework that captures the complex interplay between mental health, social relationships, and medical factors.

## The present study

The present study addresses these gaps by examining the impact of CM on biopsychosocial risks during pregnancy with an emphasis on the interactions between these factors. Using an exploratory network approach, this study aims to answer the following research questions:

- How central are the five CM subtypes within the estimated network of perinatal risk and protective factors? Centrality in a network identifies variables that are most strongly connected to the rest of the network and may therefore play a particularly important role in the overall pattern of relationships.
- Are there differential effects of CM subtypes on perinatal health? Based on previous findings, we hypothesize that emotional abuse and neglect will have the strongest effects on prenatal depression compared to other CM subtypes (Humphreys et al. 2020).

By adopting a network approach, this study aims to provide novel insights into the complex interconnections of psychological, social, and medical risk factors during pregnancy, ultimately contributing to a more holistic understanding of maternal perinatal health outcomes in individuals with a history of CM.

## Methods

### Study design

The present study is part of the *Healthy Birth* study, a randomized-controlled pilot study (described in Téoule et al. 2024). Data for this work were collected at study inclusion, before any intervention was provided to the participants. Participants received all questionnaires on their smartphone via the *BuddyCare* app (Buddy Healthcare 2021). The present analyses were pre-registered (https://osf.io/p3nsh/?view_only=87efdce20b844996af9a42660d8f88f6).

### Participants

The *Healthy Birth* study enrolled *N* = 190 participants from greater Mannheim, Germany. Nine resigned before answering any questionnaires. Of those remaining 131 provided their medical maternity records in addition to answering the questionnaires. Hence, the final sample comprised *N* = 131 pregnant individuals in pregnancy week 10–20. Demographic properties are displayed in Table 1.

**Table 1:** Sample description (*N* = 131)

	M (SD)	% (*N*)
Age	32.42 (3.77)	
Country of birth		
Germany		88.5 (116)
Other European country		7.6 (10)
Non-European country		3.8 (5)
Education		
University or university of applied sciences		63.3 (83)
Technical college		8.4 (11)
Vocational training or apprenticeship		23.7 (31)
None		4.6 (6)
Primiparous		51.1 (67)
Couple relationship		99.2 (130)

### Measures

#### **Childhood maltreatment**

*Childhood maltreatment* was measured with the German 28-item version of the *Childhood Trauma Questionnaire* (CTQ; Klinitzke et al. 2012). The CTQ consists of five subscales: physical abuse (PA), emotional abuse (EA), sexual abuse (SA), emotional neglect (EN) and physical neglect (PN), which consist of 5 items each with higher values indicating higher levels of maltreatment. Subscale scores can be classified into the severity levels “none-minimal”, “low-moderate”, “moderate-severe” and “severe-extreme” (Häuser et al. 2011). Reliability for EA was α = 0.91, for PA α = 0.90, for SA α = 0.96, for EN α = 0.92, and for PN α = 0.59.

#### **Prenatal depression**

Prenatal depression was assessed with the *Edinburgh Postpartum Depression Questionnaire* (EPDS; Cox et al. 1987). The scale consists of 10 items and higher scores indicate more depressive symptoms. Reliability in this study was α = 0.86.

#### **Prenatal anxiety**

Prenatal anxiety was assessed by the *Pregnancy-Related Anxiety Questionnaire* (PRAQ-R2; Huizink et al. 2016; Mudra et al. 2019). It consists of 10 items with higher scores indicating higher levels of anxiety. Reliability in this study was α = 0.82.

#### **Maternal-Foetal attachment**

Maternal-foetal attachment was assessed by the *Maternal-Foetal Attachment Scale* (MFAS; Cranley 1981). The MFAS consists of 24 items, a higher score indicates higher attachment. Reliability in this study was α = 0.86.

#### **Medical risk**

Information on medical risk came from participants’ official maternity records including medical data from the first routine pregnancy examination by their obstetrician/gynaecologist. This examination is usually conducted between pregnancy week 7 and 10. We assessed the following medical risk factors: age, weight, height, preexisting diabetes, preexisting severe maternal medical disorder, previous miscarriage, previous preterm delivery, previous caesarean section, previous small for gestational age baby, previous perinatal child death, previous rhesus incompatibility, previous fertility treatment and previous congenital anomaly. Risk factors were weighted by a licensed obstetrician from the study team, adapted from the Alberta Perinatal Health Program. (2014) and summed into a medical risk index with higher values indicating higher medical risk. Weighting of risk factors is displayed in Table 2.

**Table 2:** Medical risk scoring adapted from Alberta Perinatal Health Program (2014)

Condition	Score
Pre-pregnancy	
Age < 17 at delivery	1
Age > 35 at delivery	2
Weight > 91 kg	1
Weight < 45 kg	1
Height < 152 cm	1
Diabetes	3
Heart Disease	1
Hypertension	3
Chronic kidney disease	2
Other medical disorder (e.g., epilepsy, severe asthma, lupus, Chron’s disease)	1
Previous obstetric history	
Dead child (neonatal death or stillbirth)	3
Preterm delivery (20–37 weeks)	1
Abortion between 12 and 20 and < 500 g	1
Cesarean section	2
Small for date birth (5th percentile)	1
Rhesus incompatibility	1
Significant congenital anomaly (e.g., chromosomal, heart, CNS defects)	1

#### **Social support**

Participants completed the *Questionnaire on Social Support* short form (FSozU-K14; Fydrich et al. 2009). It consists of 14 items and higher values indicate higher social support. Reliability was α = 0.92.

#### **Relationship quality**

Relationship quality was assessed with the Partnership Questionnaire short form [“Partnerschaftsfragebogen”] (PFB-K; Kliem et al. 2012). It consists of 10 items with higher values indicating higher relationship quality. Internal consistency was α = 0.81.

For further analyses the sum scores of the questionnaires were used where appropriate. For the CTQ the five subscale scores were used.

### Statistical analyses

All analyses were conducted with R (R Core Team 2021) and R Studio (R Studio Team 2020). We fitted a regularized partial correlation network model (Epskamp and Fried 2018) to our data using the *qgraph* package (Epskamp et al. 2012). Included nodes were: All five CTQ subscale scores, EPDS, PRAQ-R2, FSozU-K14, MFAS and PFB-K sum scores and the medical risk score. We applied a Least Absolute Shrinkage and Selection Operator (LASSO) regularization to set small partial correlations to zero (Friedman et al. 2008). The Extended Bayesian Information Criterion, a parameter to set the degree of regularization applied to sparse correlations, was set to *γ* = 0.5 (J. Chen and Chen 2008). Stability of centrality measures and the accuracy of edge-weights were assessed by non-parametric and case-drop bootstrapping with *n* = 2500 samples using the *bootnet* package (Epskamp et al. 2018).

To answer our main research questions, we examined centrality of the five CM nodes. More precisely, we computed node strength (sum of all edges of a given node to all other nodes) and expected influence (summed weight of a node’s edges shared with remaining nodes). As a sensitivity analysis, we also estimated a network model including only EA and EN subscales of the CTQ as well as all other nodes from the original network.

## Results

Descriptive statistics of included variables are shown in Table 2. Prevalences of maternal CM are displayed in Table 3. In this sample 14.5% reported experience of one CM subtype; 10.7% reported two, 11.4% three, 3.8% four and 3.8% five subtypes. 55.7% reported not experiencing any subtype. 11% reached the cut-off of ≥ 11 on the EPDS (Levis et al. 2020).

**Table 3:** Descriptive statistics for variables included in the network analysis

	M (SD)	Min	Max
Physical abuse	5.94 (2.76)	5	25
Emotional abuse	7.74 (3.97)	5	25
Sexual abuse	5.91 (2.69)	5	22
Physical neglect	6.10 (1.98)	5	17
Emotional neglect	8.72 (4.34)	5	25
Depressive Symptoms	5.11 (4.06)	0	18
Pregnancy-related Anxiety	25.11 (7.35)	10	45
Social Support	61.95 (8.07)	33	70
Relationship Quality	21.42 (3.95)	8	27
Maternal-foetal Attachment	106.08 (20.10)	52	150
Medical risk	1.38 (1.82)	0	8

Depressive symptoms = Edinburgh Postpartum Depression Questionnaire, pregnancy-related anxiety = Pregnancy-related Anxiety Questionnaire, social support = Questionnaire on Social Support, relationship quality = Partnership Questionnaire, maternal-foetal attachment = Maternal-foetal Attachment Scale

**Fig. 1:**
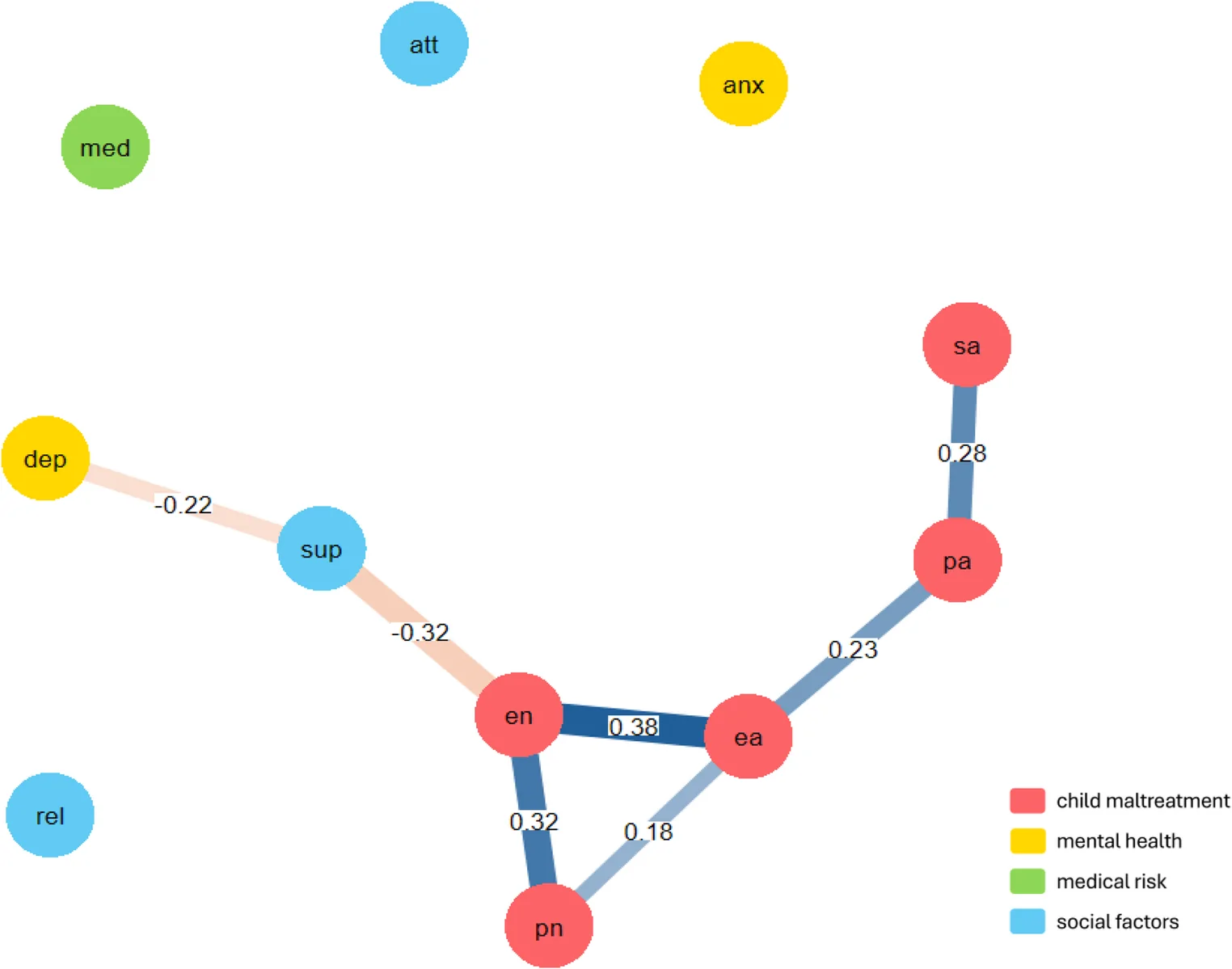
Regularized Partial Correlation Network. Note. Pa: physical abuse, ea: emotional abuse, sa: sexual abuse, en: emotional neglect, pn: physical neglect, dep: depressive symptoms, anx: pregnancy-related anxiety, sup: social support, rel: relationship quality, att: maternal-foetal attachment, med: medical risk. Nodes represent individual variables, edges represent partial correlations between variables, with edge thickness proportional to the strength of the association. Positive associations are shown in blue and negative associations in orange

The resulting network structure is displayed in Fig. 1. Case-drop bootstrapping showed correlation stability coefficients of 0.44 for both strength and expected influence, indicating acceptable stability (Epskamp et al. 2018). This indicates that the correlation between the original centrality estimates and those obtained from subsets of the data (with increasing proportions of cases dropped) remained above 0.70 even when up to approximately 44% of the sample was removed (Epskamp et al. 2018). Bootstrapped confidence intervals for all edges can be examined in the supplementary material.

**Table 4:** Prevalence of childhood maltreatment subtypes in the present study according to severity criteria (Häuser et al. 2011)

	Prevalence (%)			
	none-minimal	low-moderate	moderate-severe	severe-extreme
Emotional abuse	75.6	12.2	4.6	7.6
Physical abuse	88.5	5.3	3.1	3.1
Sexual abuse	81.7	7.6	6.1	4.6
Emotional neglect	67.9	22.1	3.8	6.1
Physical neglect	81.7	11.5	5.3	1.5

N = 131

The resulting network is relatively sparse and does not show edges between CM nodes and medical risk, maternal-foetal attachment, anxiety and relationship quality. However, it shows edges between EN and social support (*r* = -0.32). All other CM nodes are only indirectly connected to social support via EN. Social support indirectly connects CM nodes to prenatal depression (*r* = -0.22). In Table 3 strength and expected influence coefficients for all included nodes are displayed. The nodes with highest strength – i.e., connected with most other nodes – were EA (0.80) and EN (1.03). Highest expected influence – i.e., the strongest exaggerating effect on the whole network – had EA (0.80). In contrast, the most negative expected influence – i.e., the strongest buffering effect on the whole network – had social support (-0. 54).

**Fig. 2:**
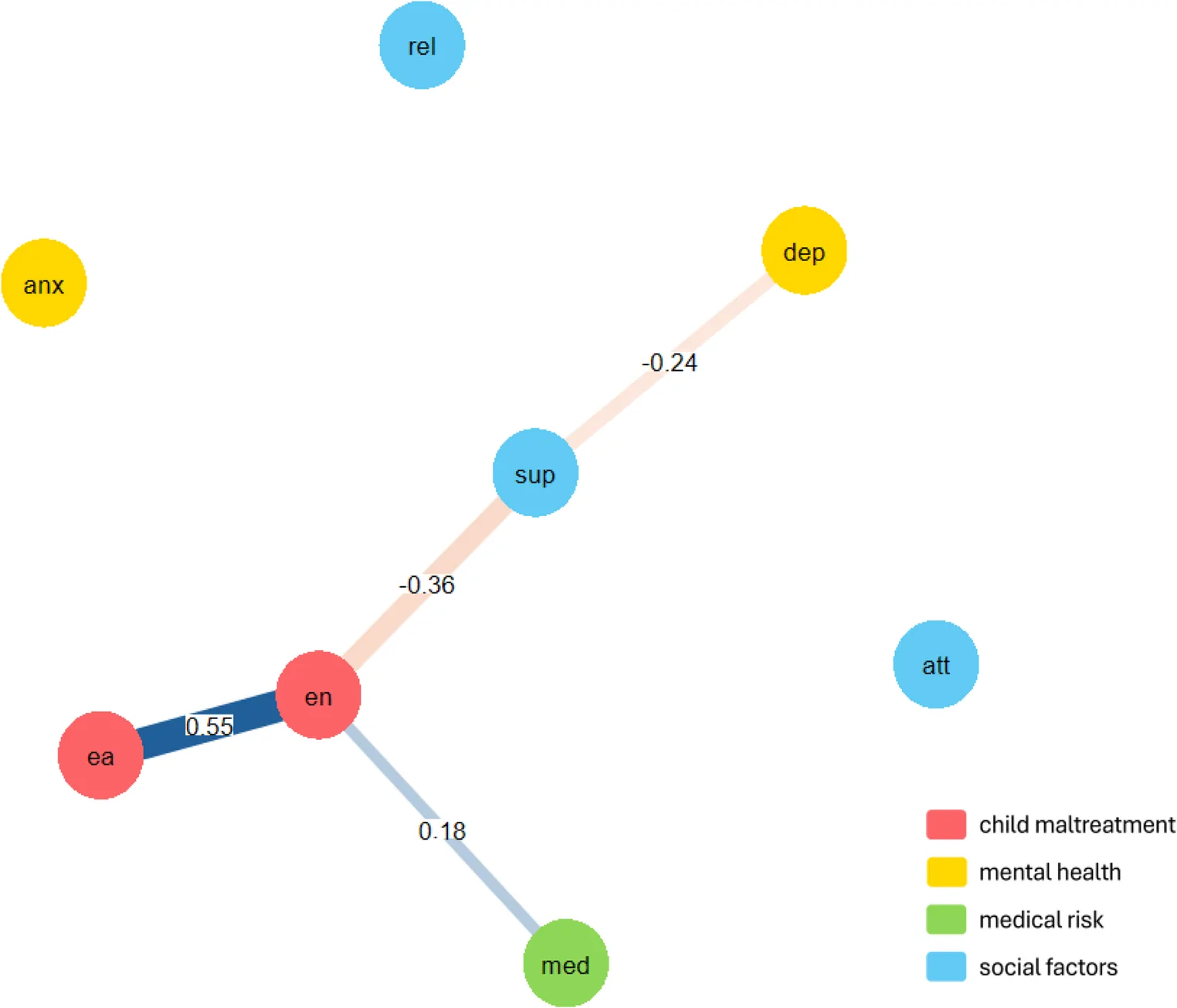
Network structure of the reduced regularized partial correlation network. Note. Ea: emotional abuse, en: emotional neglect, dep: depressive symptoms, anx: pregnancy-related anxiety, sup: social support, rel: relationship quality, att: maternal-foetal attachment, med: medical risk. Nodes represent individual variables, edges represent partial correlations between variables, with edge thickness proportional to the strength of the association. Positive associations are shown in blue and negative associations in orange.

Due to substantial correlations among CM nodes, we assessed potential collinearity using the goldbricker function (Jones 2017). The analysis showed that only 22% of correlations between EA & PA, EA & SA, and EA & PN were significantly different, falling just below the commonly recommended 25% threshold. This suggests a moderate degree of redundancy among these nodes, indicating they may not contribute sufficiently distinct information to the network. As a sensibility analysis, we fitted a more parsimonious model without SA, PA, and PN nodes, displayed in Fig. 2. Correlation stability for expected influence declined from 0.44 to 0.36, while stability for strength increased from 0.44 to 0.52. Additionally, one new edge emerged between medical risk and EN (*r* = 0.18). All other edges between CM nodes and other nodes, from EN through social support to depression, remained intact. Strength and expected influences are displayed in Table 5.

**Table 5:** Full centrality results

	Original network		Reduced network	
	Strength	Expected influence	Strength	Expected influence
Physical abuse	0.51	0.51	-	-
Emotional abuse	0.80	0.80	0.55	0.55
Sexual abuse	0.28	0.28	-	-
Physical neglect	0.51	0.51	-	-
Emotional neglect	1.03	0.39	1.09	0.38
Depressive Symptoms	0.22	-0.22	0.24	-0.24
Social Support	0.54	-0.54	0	0
Pregnancy-related Anxiety	0	0	0.59	-0.59
Relationship quality	0	0	0	0
Maternal-foetal Attachment	0	0	0	0
Medical risk	0	0	0	0

Depressive symptoms = Edinburgh Postpartum Depression Questionnaire, pregnancy-related anxiety = Pregnancy-related Anxiety Questionnaire, social support = Questionnaire on Social Support, relationship quality = Partnership Questionnaire, maternal-foetal attachment = Maternal-foetal Attachment Scale

## Discussion

We explored how different subtypes of childhood maltreatment as experienced by pregnant women relate to perinatal bio-psycho-social health by applying an exploratory network approach. Results revealed a network in which CM nodes were connected to indicators of perinatal health. Specifically, CM was associated with lower prenatal social support and indirectly with higher prenatal depressive symptoms, but not with pregnancy-related anxiety, maternal-foetal attachment and prenatal relationship quality. Medical risk was linked to CM only in a reduced network that accounted for collinearity of physical and sexual maltreatment with emotional maltreatment. Among CM subtypes, EA and EN emerged as the most central subtypes in the network. These results contribute to understanding the long-term role of CM experiences by demonstrating that CM is associated with aspects of mental, social and physical health simultaneously, even when considering interrelations between them. Thus, a history of CM is associated with biopsychosocial health risks already during pregnancy– even in a non-clinical, low-risk sample.

Regarding our main research question, our results confirm that CM does co-occur with several prenatal biopsychosocial health risks. Especially emotional subtypes of CM showed high centrality in this network. An important consideration when interpreting the centrality results is the substantial overlap between different maltreatment subtypes. Individuals exposed to one form of maltreatment often experience additional forms (Warmingham et al. 2019), resulting in high intercorrelations among subtypes. Consequently, the centrality of emotional maltreatment we found may partly reflect its shared variance with other maltreatment subtypes rather than a unique role within the network. To address this possibility, we conducted a sensitivity analysis in which other maltreatment subtypes were removed from the model. The results showed that emotional maltreatment remained similarly connected to prenatal risk factors, supporting the robustness of its central position in the network. However, even after consolidating the role of emotional maltreatment, it is important to note that high centrality does not imply causality. Thus, although emotional maltreatment emerged as particularly central, this finding should not be interpreted as evidence that it is more important or harmful than other forms of maltreatment. Instead, its central position may indicate that emotional maltreatment captures aspects of adversity that commonly co-occur with prenatal risk factors and may thus be particularly relevant for understanding how prenatal factors are interconnected. Previous meta-analytic findings underline the importance of particularly emotional maltreatment showing associations with more severe mental health problems in adulthood (Humphreys et al. 2020; Nelson et al. 2017). One possible explanation is that emotional maltreatment may impair emotion awareness and regulation (Ditzer et al. 2023; Gruhn and Compas 2020) and reduce interoception and trust in one’s own body (Ditzer et al. 2025a, b). These factors are particularly relevant during the peripartum period, a time marked by pronounced physical and emotional changes.

Contrary to our hypotheses, there was no *direct* association between emotional CM and depressive symptoms. Importantly, this finding does not suggest that CM and depression are unrelated on a bivariate level─ this relationship has been consistently demonstrated in previous research (Christie et al. 2018; Humphreys et al. 2020; Racine et al. 2021). Rather, it implies that, in our sample, the association between CM and prenatal depressive symptoms is accounted for by the association of both with social support. This highlights social support as a meaningful construct for understanding prenatal health and as a potential target for prevention and intervention. As our community sample consisted mainly of highly-educated participants with low-risk pregnancies and only 11% reached the EPDS clinical cut-off, our findings cannot be generalized to clinical populations. In psychiatric populations **s**tronger associations between CM and depressive symptoms have been observed (Nelson et al. 2017). Nonetheless, social support exhibiting the most buffering effect on the total network, emphasizes the importance of supportive social relationships in the perinatal period. This strengthens previous findings showing that strong social relationships can protect pregnant women from prenatal mental health difficulties (Henriksen et al. 2015; Pilkington et al. 2015; Røsand et al. 2011) and contribute to a more positive pregnancy and childbirth experience (Hoffmann et al. 2023).

Notably, although previous research demonstrated associations of CM with prenatal anxiety, relationship quality and maternal-foetal attachment, no corresponding edges emerged in our network. This absence of edges does not necessarily indicate a complete lack of association on a bivariate level. Our results suggest that, within this sample, the associations between CM and these factors were small enough to be eliminated through LASSO-regularization and thus most likely of low clinical relevance. From these results we can conclude that among healthy pregnant women in early pregnancy, CM is primarily associated with social support, showing only an indirect relationship with depressive symptoms. Additionally, the edge between EN and medical risk emerged only after excluding non-emotional maltreatment nodes, suggesting this association may have been previously suppressed due to shared variance with the removed CM subtypes. When interpreting these results, it should be noted that, although validated in the Alberta Perinatal Health Program (2014), the risk score was not specifically validated in this sample. Another factor that may attenuate potential association is the timing of the medical risk assessment, which occurred early in pregnancy in our study (weeks 7–10), before routine screenings for conditions such as gestational diabetes or foetal anomalies typically takes place in Germany (around weeks 20–22). Especially these later-emerging complications are particularly prevalent among individuals with a history of CM (Mamun et al. 2023; Souch et al. 2022). Thus, findings regarding medical risk should be interpreted with caution. Future research should explore if medical risks associated with CM may develop only later in pregnancy, whereas its effect on mental health and social relationships may become apparent earlier in pregnancy, as suggested by our results.

### Strengths and limitations

One main strength lies in the study design enabling simultaneous analysis of multiple risk/protective factors and offering a holistic bio-psycho-social understanding of prenatal women’s health. Importantly, the study differentiates between CM subtypes, capturing the often-overlooked heterogeneity of these experiences. Despite its strengths, this study has several limitations. First, the cross-sectional design limits causal inference. However, given the temporal precedence of CM nodes relative to the other variables, edges originating from CM nodes may be interpreted as CM affecting other variables. Second, most measures were based on self-report, which may introduce bias due to social desirability. Third, reliability of physical neglect was low, which might limit conclusions on this subtype. This phenomenon was previously found in literature and is handled differently throughout studies (discussion in Ditzer et al. 2025a, b). As many experts argue that capturing experiences of physical neglect is still necessary, despite the heterogeneity of the concept, we chose to include it in the final network while interpreting results carefully. Finally, the community-based sample included participants with minimal CM exposure and was predominantly well-educated. This may restrict the generalizability of findings to more diverse or clinical populations. Another limitation involves correlations between CM subtypes. Although their overlap reflects real-world co-occurrence, it complicates network estimation. Regularization methods like LASSO suppress shared variance, highlighting only the strongest distinct associations and potentially obscuring others. Nevertheless, given our goal to identify the most robust links, this exploratory approach is valuable.

### Implications

The present findings have important implications for prevention in prenatal care. Our results show that even in a low-risk community-based sample CM is closely linked to biopsychosocial health - even in early pregnancy. Prenatal care providers should consider that CM is associated with increased psychosocial vulnerability in pregnant women. Midwives and gynaecologists should therefore routinely assess CM as it may give important information on the co-occurence of potential risk factors which need to be addressed to prevent greater difficulties in the transition to parenthood. Targeting perceived social support could be a key protective factor against depression in the presence of CM. This could be fostered by involving pregnant women’s social networks, educating peers about their role in providing support, or helping women establish supportive relationships where they are lacking. Indicated prevention for individuals with CM should not only promote access to existing services but may also require additional time to build trust, particularly among those with histories of emotional maltreatment. Policy initiatives are essential to ensure sustained funding and structural resources, enabling practitioners to provide the time and support necessary for this vulnerable population.

## Supplementary Information

Below is the link to the electronic supplementary material.

Supplementary Material 1

## Data Availability

The data that support the findings of this study are not openly available due to reasons of sensitivity of medical data and are available from the corresponding author upon reasonable request. Data are located in controlled access data storage at Medical Faculty Mannheim, University of Heidelberg.
