# Supplementary material for: Mapping links between childhood maltreatment and biopsychosocial health in pregnancy: a network analysis
Source: Arch Womens Ment Health. 2026 Aug 19;29(5):114. doi: 10.1007/s00737-026-01749-4 (PMC13486009; doi:10.1007/s00737-026-01749-4)
Supplement: Supplementary file 1 [file 737_2026_1749_MOESM1_ESM.docx]

**Figure S1**

*Bootstrapped Confidence Intervals*
